# Empowering Data Sharing and Analytics through the Open Data Commons for Traumatic Brain Injury Research

**DOI:** 10.1089/neur.2021.0061

**Published:** 2022-04-05

**Authors:** Austin Chou, Abel Torres-Espín, J. Russell Huie, Karen Krukowski, Sangmi Lee, Amber Nolan, Caroline Guglielmetti, Bridget E. Hawkins, Myriam M. Chaumeil, Geoffrey T. Manley, Michael S. Beattie, Jacqueline C. Bresnahan, Maryann E. Martone, Jeffrey S. Grethe, Susanna Rosi, Adam R. Ferguson

**Affiliations:** ^1^ Brain and Spinal Injury Center, University of California San Francisco, San Francisco, California, USA.; ^2^ Department of Neurological Surgery, University of California San Francisco, San Francisco, California, USA.; ^3^ San Francisco Veterans Affairs Healthcare System, San Francisco, California, USA.; ^4^ Department of Physical Therapy and Rehabilitation Science, University of California San Francisco, San Francisco, California, USA.; ^5^ Weill Institute for Neuroscience, University of California San Francisco, San Francisco, California, USA.; ^6^ Kavli Institute of Fundamental Neuroscience, University of California San Francisco, San Francisco, California, USA.; ^7^ Department of Radiology & Biomedical Imaging, University of California San Francisco, San Francisco, California, USA.; ^8^ Department of Anesthesiology, University of Texas Medical Branch at Galveston, Galveston, Texas, USA.; ^9^ Moody Project for Traumatic Brain Injury Research, University of Texas Medical Branch at Galveston, Galveston, Texas, USA.; ^10^ Department of Neuroscience, University of California San Diego, San Diego, California, USA.

**Keywords:** data sharing, FAIR principles, multi-variate analysis, Open Data Commons, principal component analysis, traumatic brain Injury

## Abstract

Traumatic brain injury (TBI) is a major public health problem. Despite considerable research deciphering injury pathophysiology, precision therapies remain elusive. Here, we present large-scale data sharing and machine intelligence approaches to leverage TBI complexity. The Open Data Commons for TBI (ODC-TBI) is a community-centered repository emphasizing Findable, Accessible, Interoperable, and Reusable data sharing and publication with persistent identifiers. Importantly, the ODC-TBI implements data sharing of individual subject data, enabling pooling for high-sample-size, feature-rich data sets for machine learning analytics. We demonstrate pooled ODC-TBI data analyses, starting with descriptive analytics of subject-level data from 11 previously published articles (*N* = 1250 subjects) representing six distinct pre-clinical TBI models. Second, we perform unsupervised machine learning on multi-cohort data to identify persistent inflammatory patterns across different studies, improving experimental sensitivity for pro- versus anti-inflammation effects. As funders and journals increasingly mandate open data practices, ODC-TBI will create new scientific opportunities for researchers and facilitate multi-data-set, multi-dimensional analytics toward effective translation.

## Introduction

Traumatic brain injury (TBI) is a leading cause of neurological disorders and affects >69 million persons annually worldwide.^1,2^ Incidence of TBI is expected to rise each year, and >3 million patients in the United States alone and many more globally suffer from chronic TBI-related disabilities.^2–4^ Despite the abundance of pre-clinical TBI studies, randomized controlled clinical trials have consistently failed.^5,6^ One significant challenge for the development of effective treatments is the heterogeneity of injuries and the varied pathological biology captured by the broad definition of TBI: a disruption of neurological function caused by a bump, blow, or jolt to the head or penetrating head injury.^7^

Biological injury responses can differ dramatically across injury sites, injury severities, and patient characteristics.^8^ To capture the heterogeneity of clinical TBIs, a multitude of pre-clinical TBI models have been developed to isolate specific injury mechanisms.^9^ Although the diverse injury parameters and outcome measures used by different experimenters do effectively recapitulate distinct aspects of clinical pathology, the breadth of pre-clinical models and research ultimately makes inferential insights difficult to compare across studies and translate across species. Pre-clinical TBI models have thus largely been treated as very distinct representations of clinical TBI, circumventing the complexity of TBI heterogeneity instead of directly addressing it. However, the wealth of data collected across TBI models presents a new opportunity for rigorous joint analyses across studies and across pre-clinical TBI models to directly investigate common biological features underlying heterogeneity.

Indeed, there is growing interest and support for the application of Big Data frameworks and multi-dimensional machine learning to TBI research.^10–12^ Such techniques have been recently used with clinical data to reveal TBI pathophysiology persistent across heterogeneous patients.^13,14^ Whereas similar efforts in pre-clinical TBI research are still nascent, they represent a unique perspective toward unraveling common pathological mechanisms and bridging pre-clinical to clinical research.

A major obstacle to the Big Data approach is the underdeveloped and -utilized practice of data sharing and data standardization and harmonization in the pre-clinical TBI field. Clinical TBI data programs, such as Transforming Research and Clinical Knowledge in TBI (TRACK-TBI) and Collaborative European NeuroTrauma Effectiveness Research in TBI (CENTER-TBI), have dramatically improved access to data and enabled multi-dimensional analytics in clinical research.^15–18^ In contrast, most pre-clinical TBI data and research have been communicated and shared solely through publications without the release of the underlying data. The data of each published specimen are thus sequestered as summarized aggregates, which makes individual subject-level data inaccessible for data reuse and further analytics.^12,19^

Additionally, the language and terminology of collected variables can differ in name and definition between labs. The National Institute of Neurological Disorders and Stroke (NINDS) have released dictionaries of Common Data Elements (CDEs), basic units of data that prescribe the data type and standardize the language for variables in an effort to improve the reproducibility of clinical and pre-clinical TBI research.^20,21^ However, there remains an unmet need for open data infrastructures that host pre-clinical TBI data and for data sets to begin integrating the NINDS-defined CDEs for data sharing and reusability.

In this article, we present the Open Data Commons for TBI (ODC-TBI), a platform and repository for data sharing for the global pre-clinical TBI research community. The infrastructure is developed in collaboration with the Neuroscience Information Framework (NIF).^22^ Building upon previous work on the Open Data Commons for Spinal Cord Injury (ODC-SCI),^23,24^ we developed the ODC-TBI for protected data sharing while upholding data stewardship principles toward making biomedical data Findable, Accessible, Interoperable, and Reusable (FAIR).^25^ To jumpstart FAIR sharing in pre-clinical TBI, we standardized data sets from 11 publications along NINDS-defined CDEs and uploaded them to the ODC-TBI. As a proof of concept for Big Data analytics enabled by the ODC-TBI, we aggregated data from three separate experiments uploaded to the ODC-TBI and harnessed multi-variate analytics to uncover persistent patterns of inflammatory response in the controlled cortical impact TBI mouse model. Altogether, we illustrate the infrastructure of the ODC-TBI to promote data sharing within the pre-clinical TBI research community and demonstrate the utility of multi-data-set, multi-dimensional analytics to uncover common TBI pathophysiology across heterogeneous experimental features.

## Methods

### Data formatting and upload to the Open Data Commons for Traumatic Brain Injury

Data from 11 published studies at University of California San Francisco (UCSF) were collected from various data sources and structured according to the Tidy data format.^26^ Variable names were aligned to NINDS pre-clinical TBI CDEs when possible.^20,21^ Data were uploaded after the ODC-TBI data upload workflow. The specific variables and data analyzed in this article will be published and made accessible on the ODC-TBI.

### Data summarization and missing values visualization

Data sets were downloaded from the ODC-TBI and aggregated using the open-source programming language, R.^27^ Data summaries were generated using *tidyverse*^28^ for data-frame manipulation and *ggplot2*,^29^ *RColorBrewer*,^30^ and *colorRamps*^31^ R packages for visualization.

Missing values visualizations were generated using the “vis_miss” function in the *naniar* R package.^32^ Labeling of the types of missingness was done manually by relying on researcher familiarity with the data set.

### Multi-dimensional use case workflow

Quantitative polymerase chain reaction (qPCR) measures of six cytokines (interleukin 1-beta [IL-1β], tumor necrosis factor alpha [TNF-α], inducible nitric oxide synthase [iNOS], *Ym1* chitinase-like protein [Ym1], cluster of differentiation 206 [CD206], and transforming growth factor beta [TGF-β]) from three experiment cohorts^33,34^ were combined into a single data set. A missing-values visualization was generated using the *naniar* R package, and rows that were missing values across all cytokine variables (i.e., columns) were removed (one row removed). Little's missing completely at random (MCAR) test was performed using the “LittleMCAR” function in the *BaylorEdPsych* R package^35^ to determine the pattern of missingness to meet statistical assumptions.^36^

To impute missing values, we used the “mice” function in the *mice* R package^37^ with the parameters: 10 imputations, predictive mean matching method, and a seed value of 200. Linear principal component analysis (PCA) was performed using the “prcomp” function in the *stats* R package^27^ with centering and scaling.^38,39^

To correct for the batch effect (i.e., effect of different studies), we added a z-score standardization step after removing the rows missing data across all columns and before data imputation. We first calculated the mean and standard deviation for each cytokine for each of the three studies. For each cytokine data point, we then subtracted the respective mean and divided by the standard deviation of the study.

Principal components (PCs) were retained using classic tools from the factor analysis tradition: 1) scree plot, 2) Kaiser rule (eigenvalue, >1), and 3) PC determination based on examination of loading saturation. In addition, we performed iterative testing of accuracy/stability of PC patterns under imputation iterations. To determine the stability of the PCA results across the 10 imputations generated through *mice*, we utilized the “component_similarity” function in the *syndRomics* R package.^40^ We reported the resulting Congruence Coefficient, Cattell's salient similarity metric, and root mean square error (RMSE). Because the PCA outputs for each imputation were highly similar, we averaged all 10 imputations together to generate the final imputed data set. PCA was then performed on the imputed, averaged data set for further analysis.

To visualize the scree plot, we calculated the variance accounted for (VAF) for each PC from the “sdev” output of “prcomp”:

$$VAF_{i}=sdev_{i}^{2}∕\Sigma \left(sdev^{2}\right)$$

where *i* is the PC number and the denominator is the sum of the variance across all PCs. We selected the top PCs that collectively explained >80% of the variance in the data and had biological interpretations. PC loadings were calculated and visualized using the “syndromic_plot,” “barmap_loading,” and “heatmap_loading” functions in the *syndRomics* R package. PC scores were obtained from the “x” output of “prcomp,” which transformed the original variables into values along each PC.

To determine the study and injury effects and injury and age effects, we performed a two-way analysis of variance (ANOVA) with Tukey's honestly significant difference HSD *post hoc* using the “aov” and “TukeyHSD” functions in the *stats* R package, respectively.

To compare effect sizes and observed power, we performed two-way ANOVA for the main effects and interaction of Injury and Age on PC1 and PC2 of adult and aged sham animals and animals at 7 days post-injury (dpi) from the aggregated data set (*n* = 47). We additionally filtered for the Chou and colleagues 2018 cohort (*n* = 31) and performed two-way ANOVA on the six individual inflammatory markers. The effect size (η^2^) of the Injury effect, Age effect, or interaction was calculated from the ANOVA *F* table as:

$$\eta^{2}=\frac{termsumofsquares}{totalsumofsquares}$$

To obtain observed power, we calculated the partial *η^2^*:

$$partial\eta_{0}^{2}=\frac{termsumofsquares}{\left(termsumofsquares+residualsumofsquares\right)}$$

We then converted the partial *η^2^_0_* (which is based on sample estimates) to the partial *η^2^* based on Cohen's *f* according to the G*Power manual^41^:

$$partial\eta^{2}=\frac{partial\eta_{0}^{2}*\left(N-k\right)}{\left(N-k*partial\eta_{0}^{2}\right)}$$

where *N* is the total number of samples and *k* is the total number of groups in the experimental design. Partial *η^2^* was converted to Cohen's *f*:

$$f=\sqrt{\frac{partial\eta 2}{1-partial\eta 2}}$$

The observed power was then calculated from Cohen's *f* using the “pwr.f2.test” function in the *pwr* R package^42^.

## Results

### Open Data Commons for traumatic brain injury infrastructure for data sharing and security

**FIG. 1.**
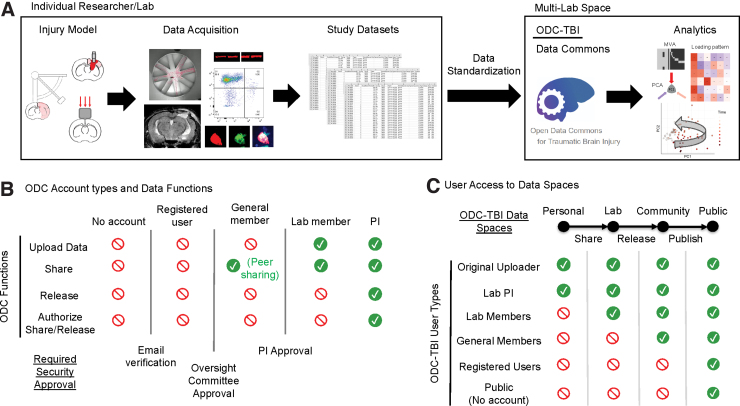
Open Data Commons for Traumatic Brain Injury (ODC-TBI) data flow and accessibility summary. (**A**) Experiments are currently carried out by individual researchers and labs. Resulting data sets are commonly preserved within lab resources (e.g., hard drives, lab notebooks, etc.). The ODC-TBI provides documentation and guidance to help standardize data sets with respect to NINDS-defined pre-clinical Common Data Elements (CDEs). Data sets from multiple labs and centers can then be uploaded into the ODC-TBI and shared and combined for further analysis. (**B**) The ODC-TBI has five user types with three steps for security. Each user type has different available functions on the site. After an e-mail verification and approval by the ODC-TBI committee to ensure that the user is a researcher in the TBI field, they become a general member. They can then join or create a lab, which requires the lab PI's approval. Lab members can upload and share their data within the lab they have joined. Last, PI-level users can also initiate the data set release/publication process, increasing the accessibility of their data to others outside of their lab. (**C**) The ODC-TBI consists of four Data Spaces. Each Data Space has different levels of accessibility. Data sets are delegated to a Personal space when they are first uploaded; Personal data sets are accessible only to the uploader and their lab PI. Data sets are shared into the Lab space where they can be accessed by anyone in the lab. Data sets can be released into the Community space where other general members can access them. Last, PIs can publish their data sets, which will make the data set accessible to the general public as citable units of research with unique digital object identifiers. NINDS, National Institute of Neurological Disorders and Stroke; PI, Principal Investigator.

The purpose of the ODC-TBI is to establish an infrastructure to facilitate effective data-sharing practices within the pre-clinical TBI research community and expand the data standardization and harmonization guidelines initiated by the NINDS.^20,43^ Additionally, the ODC-TBI interface has been developed to address the concerns of pre-clinical TBI researchers toward data-sharing practices^21^ and empower the researchers through an intuitive interface. Currently, the ODC-TBI provides guidelines to help researchers format their data set according to best practices for data interoperability^26,44^ and standardize them according to FAIR principles^25^ and NINDS-defined CDEs. Once prepared, data sets can be uploaded to the ODC-TBI and then further combined for Big Data analytics (Fig. 1A).

Protecting a lab's data from misuse by third parties is a major concern of investigators.^45,46^ To address this obstacle, the ODC-TBI is built on a robust cloud-based cyberinfrastructure through the California Institute for Telecommunications and Information Technology, which includes e-commerce–grade security and encryption. In addition, ODC-TBI has established several approval protocols to provide qualified access to sensitive data while enabling open access to published data (Fig. 1B). Uploading, sharing, and accessing data are only possible for users who have a verified institutional e-mail and have been approved for lab membership by a Principal Investigator (PI) with a lab in ODC-TBI. The process of data sharing requires authorization by the PI, and the PI can remove data sets from the shared space at any time.

When data are first uploaded, it is restricted to a Personal space accessible to only the original uploader and their lab PI. Once approved by the PI, the data set migrates to the Lab space where others in the same lab will be able to access the data set. The PI can approve the release of a data set into the Community space where other members of the ODC-TBI community will be able to access the data set. Last, the PI can trigger a data-set publication process on the ODC-TBI; once completed, the data set will be published as a citable unit of research with a unique digital object identifier (DOI) and made accessible to the general public (Fig. 1C). By granting the PI full control of their data sharing at all times and requiring multiple security checks, we can alleviate security concerns regarding data sharing.

Another common obstacle toward data sharing is the lack of guidance toward adequately organizing the data.^47^ Experimental data are commonly stored on spreadsheets with various structures that strive to make the data clearly readable by humans. This includes nested labels, different font sizes, and multiple tables on the same spreadsheet representing different parameters (Supplementary Fig. S1A). However, though this approach makes data easy to understand to the original experimenters, the practice creates wide variations in data formats and presents an intractable problem for large-scale data harmonization, interoperability, and merging. The ODC-TBI requires that data sets be reformatted into the Tidy format, a standardized data format ideal for data storage, aggregation, and multi-data-set analytics (Supplementary Fig. S1B).^26^ The ODC-TBI contains written tutorials to guide researchers in formatting their data into the Tidy structure. We also encourage the upload of data-set–associated data dictionaries (Supplementary Fig. S1C). Data dictionaries help provide critical definitions for each variable in the data set, essential information, such as the unit of measurement and additional comments about the experimental protocol, that improve the interpretability and reusability of the data set (e.g., reasons for excluding samples).

**FIG. 2.**
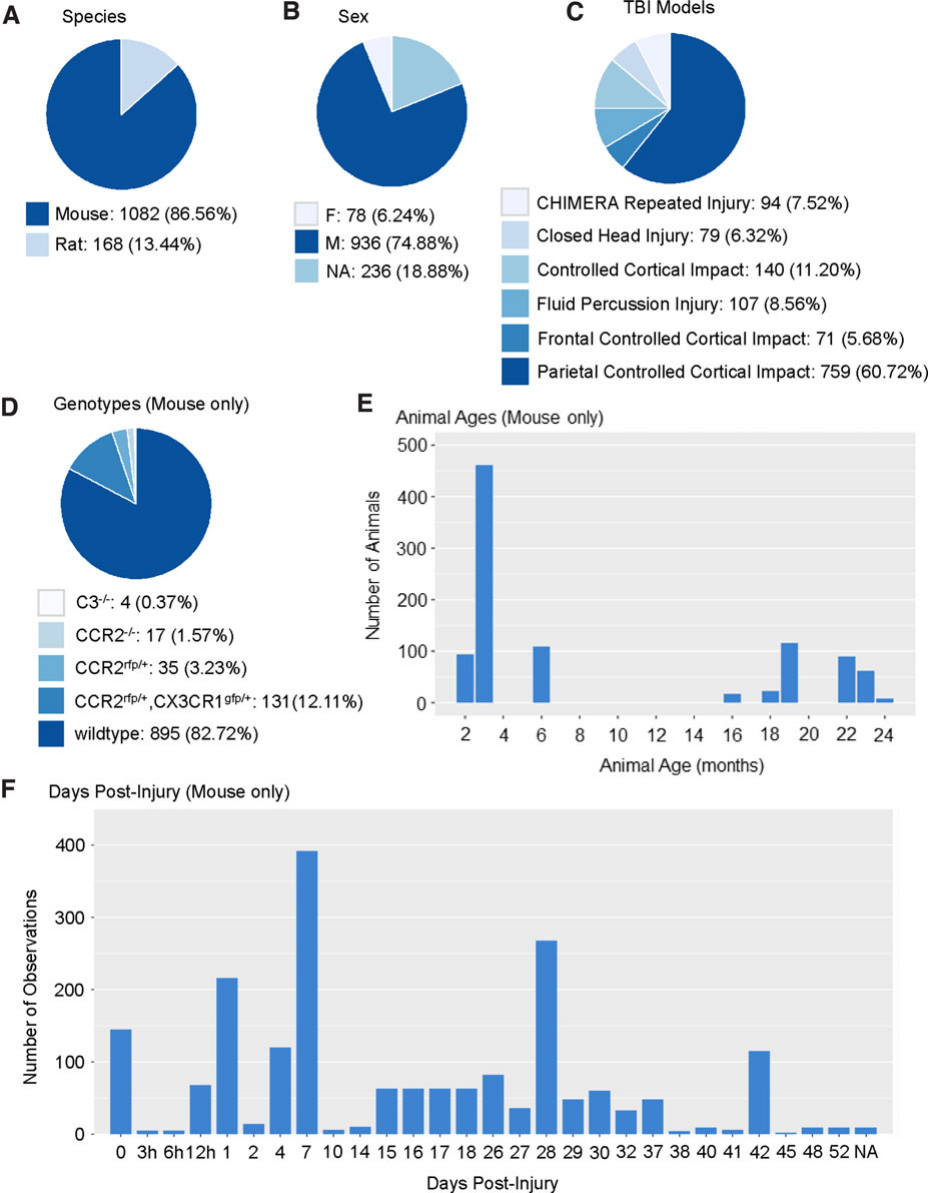
Descriptive summaries of data aggregated from 11 pre-clinical TBI publications from the UCSF on the ODC-TBI. (**A**) The 11 data sets constituted data from 1250 unique animals, with the majority being mice. (**B**) The majority of subjects were male, with a small proportion of female, animals. Notably, 18.9% of the subjects were missing records of male or female. (**C**) The primary TBI model utilized was the controlled cortical impact model with the greatest representation by parietal injuries. There were also a smaller number of fluid percussion injury subjects, closed TBI subjects, and repeated closed-TBI models using the CHIMERA impactor. (**D**) Of the mice subjects, the predominant genotype was wild type. The remaining mouse models included C3-knockout, CCR2-knockout, CCR2-rfp transgenic, and CX3CR1-gfp and CCR2-rfp transgenic animals. These transgenics reflected the interest in inflammatory pathways after TBI in the publications. (**E**) Mice subjects' age at time of injury showed a bimodal distribution encompassing young (2–6 months) and old (16+ months) animals. Age distribution reflected the focus on the effect of aging on TBI processes. (**F**) Data were collected at a variety of time points from the mice experiments. Time points with the greatest number of observations were 0 days post-injury (dpi), 1 dpi, 7 dpi, and 28 dpi. The breadth of time points reflected time-course studies as well as the interest in both acute and chronic effects of TBI in the studies. C3, complement C3; CCR2, C-C motif chemokine receptor 2; CX3CR1, C-X3-C motif chemokine receptor 1; CHIMERA, closed-head impact model of engineered rotational acceleration; F, female; M, male; NA, not applicable; TBI, traumatic brain injury; UCSF, University of California San Francisco.

To demonstrate the ODC-TBI, we uploaded and aggregated 11 data sets corresponding to 11 past publications from several labs at the UCSF.^33,34,48–56^ Additionally, we included an external data set from a pooled analysis from the University of Texas Medical Branch published through the ODC-TBI and reused under a creative commons (attribution) license (CC-BY 4.0).^57^ The number of animals across all 12 data sets totaled *N* = 1250 individual subjects. Data sets were harmonized according to National Institutes of Health/NINDS CDEs for pre-clinical TBI, which enabled merging of the data sets for multi-data-set descriptive analytics as presented in Figure 2. The majority of the uploaded data corresponded to mouse experiments (86.56%). The rest corresponded to rat experiments (13.44%; Fig. 2A). Overall, 74.88% of subjects were male, whereas 6.24% were female, animals. Notably, 18.88% of records were missing a value for the sex parameter as a result of irrecoverable records, which is a common issue when collecting data sets from older publications (Fig. 2B).^58^ The majority of the experiments utilized the controlled cortical impact contusion injury model (77.6%) with a smaller number of fluid percussion injury (8.56%), closed TBI (6.32%), and closed-TBI model of engineered rotational acceleration repeated injury models (7.52%; Fig. 2C).

We further visualized the characteristics of the 1082 mouse subjects. Whereas most of the mice were wild type, 17.28% of subjects were transgenic for immunology-related genes, which highlights the fact that the summarized studies were primarily focused on immunological processes of TBI (Fig. 2D). Subject age distribution showed a bimodal distribution, with most animals falling below 6 months or above 18 months of age, reflecting the nature of the studies investigating the effects of age on TBI biology (Fig. 2E). Last, a variety of acute and chronic time points were represented in the data sets (Fig. 2F). Notable peaks in time-point distribution included time 0 (often a control time point for uninjured animals), 1 dpi, 7 dpi, and 28 dpi to measure acute, subchronic, and chronic effects of TBI, respectively.

### Missing data in data structure

While working with users to prepare their data sets for upload, we observed that users often had questions regarding uploading files that contain empty cells, also termed “missing values.” Missing values are to be expected: A single data set can contain data from multiple studies with different outcome measures, resulting in a patchwork of missing and present data. Missing values analysis (MVA) is an established statistical subfield that involves descriptive statistical diagnosis of missingness patterns, such as whether data are missing completely at random (MCAR), missing at random (MAR), or missing not at random (MNAR).^58,59^ Identifying the pattern and reasons for missing data is critical for appropriate data imputation—the statistical practice of replacing missing values with plausible substitute values usually derived from the rest of the data—and multi-dimensional analytics.^58,59^ Data-set–associated methodology and data dictionary documents on the ODC-TBI can be utilized to inform MVA.

**FIG. 3.**
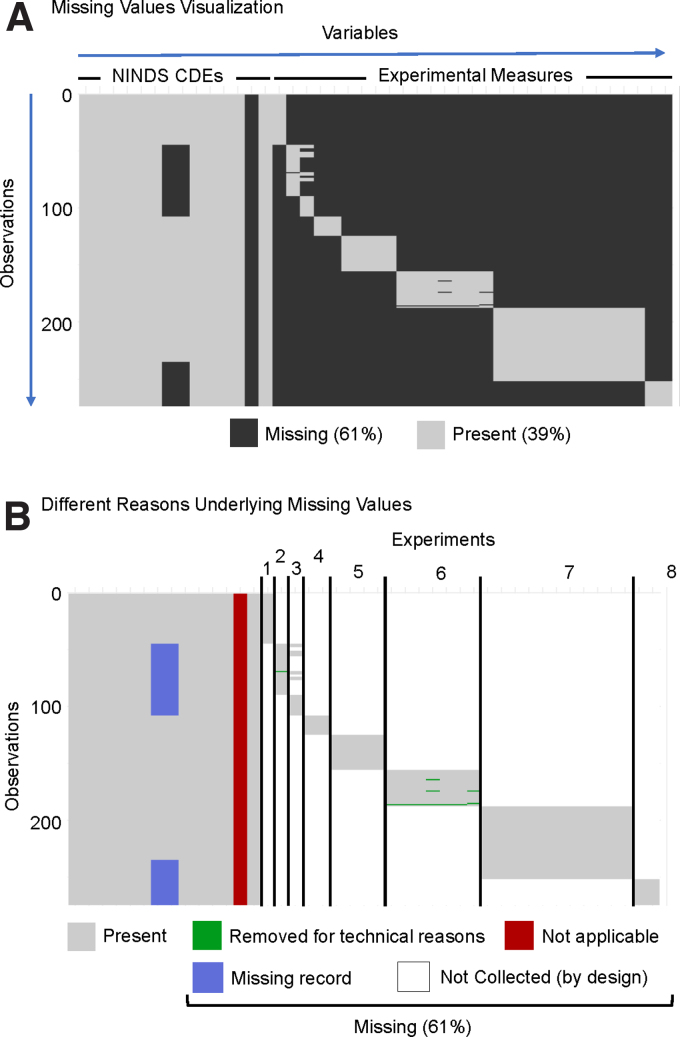
Missing value visualizations of Chou and colleagues (2018).^33^ (**A**) Typical missing value visualization shows which elements (i.e., cells) contain a value and which do not, which are thus termed missing. The uploaded data showed generally low missingness for variables (i.e., columns) corresponding to NINDS CDEs and fairly high missingness for variables corresponding to collected experimental measures. Each row corresponded to an observation, in this case a single animal subject. (**B**) Types of missingness were manually color-coded based on the type of missingness. The majority of the missing values were “Not Collected (by design)”; the data set constituted eight separate experiments, and experimental outcomes were specifically collected for subjects belonging to one experiment. The result was an extremely sparse data set by design. Another source of missingness was when a variable is “Not applicable,” which we expect in cases when a NINDS-defined CDE is not applicable to the study design. In this example, no treatments were given, so the treatment CDE column was entirely missing values. Data could also be irrecoverable because of “Missing records,” such as the subject's sex in this example and as reflected in Figure 2B. Last, data from experiments could also have been “Removed due to technical reasons.” CDEs, Common Data Elements; NINDS, National Institute of Neurological Disorders and Stroke.

Here, we highlight common reasons for missingness using the Chou and colleagues 2018 data set given the researchers' familiarity with the data set and the breadth of reasons for missingness represented.^33^ The simplest visualization for MVA recodes the data-set elements (i.e., spreadsheet cells) with a binary code (0 = missing, 1 = present) and produces a plot of black and gray for missing elements and present elements, respectively. MVA revealed that 61% of the elements in the selected data set are missing values (Fig. 3A).

Reasons for missingness can be quite varied (Fig. 3B). Most commonly, measures might not be collected at all as part of the experimental design (“not collected [by design]” white cells in Fig. 3B). For example, a sample can be used either for immunohistochemistry or for flow cytometry, but not both. Accordingly, two separate cohorts of animals are required: one planned for immunohistochemistry measures and one for flow cytometry. Conversely, there are times when an attempt is made to collect the data, but the data are excluded because of technical reasons (“removed for technical reasons” green cells in Fig. 3B). Understanding the circumstances for which the data were removed is critical for the process of data imputation.

In some cases, a variable (i.e., column) may exist in the data set, but not actually be applicable, thus leading to an entire column of missing values (“not applicable” red cells in Fig. 3B). This can also be the result of data harmonization and aggregation when certain columns are not applicable to specific data sets. In the Chou and colleagues 2018 data set, there is a column for the “treatment” CDE. However, no treatments were administered in any of the experiments, and, accordingly, the entire column is missing given that the parameter was not applicable to the data set.

Another possible reason for missing values is that the data were not recorded or were unable to be recovered from past records (“missing record” blue cells in Fig. 3B). In the Chou and colleagues 2018 data set, some subjects are missing the sex variable, which corresponds with Figure 2B. In this case, the experimental records that we collected the data from did not have the sex information readily available.

### Multi-dimensional analytics use case

To demonstrate the multi-data-set analytic workflow facilitated by the ODC-TBI, we aggregated data from three controlled cortical impact studies (i.e., independent experimental cohorts of animals; *N* = 99) published in Chou and colleagues [2018] and Morganti and colleagues [2015]^33,34^). These studies were chosen because basic multi-variate approaches require common variables between data sets. The selected studies included an injury time-course study, an aging study, and a treatment study that isolated innate immune cells from injured brain tissue, and all measured the expression of the following six inflammatory markers by qPCR: IL-1β, TNF-α, iNOS, Ym1, CD206, and TGF-β. Using the aggregated data, we performed 1) MVA, 2) missing data imputation, 3) PCA, and 4) syndromic visualization to identify salient multi-dimensional patterns of immune activation across the studies.

**FIG. 4.**
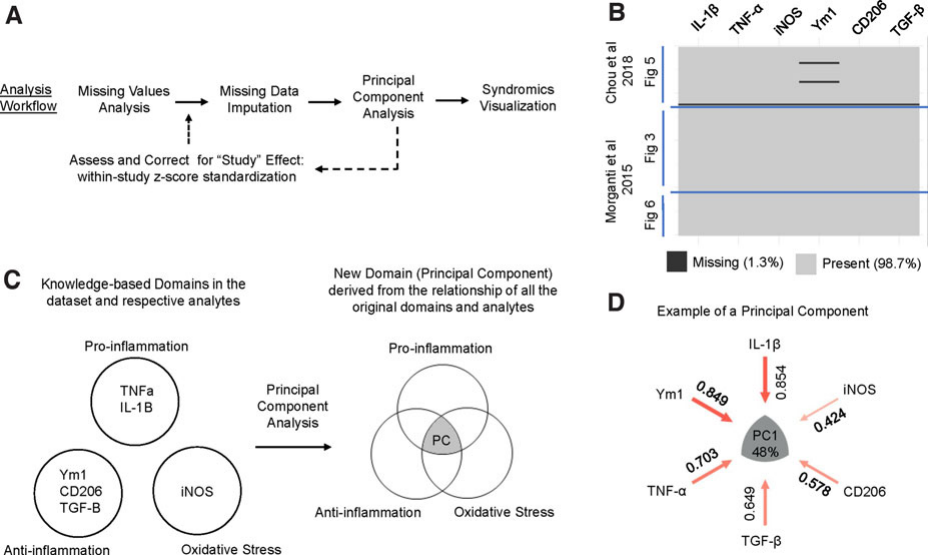
Multi-dimensional analytics use case. (**A**) We implemented an analysis workflow including missing values analysis, missing data imputation, principal component analysis (PCA), and syndromic visualizations. After an initial analysis, we implemented an additional z-score standardization step before data imputation to correct for a study effect. (**B**) Data were aggregated from three experiments (figures) from two articles: Chou and colleagues (2018) and Morganti and colleagues (2015).^33,34^ Visualization of the missing data show that 1.3% of the data set was missing values. Notably, one entire row was entirely missing, and the other two missing values were from the Ym1 variable. (**C**) Conceptual representation of PCA. The original variables (TNF-α, IL-1β, Ym1, CD206, TGF-β, and iNOS) can be categorized into the domains of proinflammation, anti-inflammation, and oxidative stress based off of existing knowledge. PCA is an unsupervised method that captures the underlying relationship between the variables—and thus the relationship between the represented knowledge domains—to derive new latent cross-domain features from the data. (**D**) The derived PC can be represented as a syndromic plot that visualizes the contributions (i.e., loadings) of each variable to the PC. Further, the PC captures a portion of the variance in the data, which is reflected by the percentage value in the center of the syndromic plot. In the example PC, 48% of the variance in the data set was accounted for, and all six of the variables were loading positively. CD206, cluster of differentiation 206; IL-1β, interleukin 1 beta; iNOS, inducible nitric oxide synthase; PC, principal component; TGF-β, transforming growth beta; TNF-α, tumor necrosis factor alpha; Ym1, *Ym1* chitinase-like protein.

After the initial analysis, we included an additional within-study z-score standardization step between MVA and missing data imputation in order to correct for an observed batch effect of study (i.e., study effect; Fig. 4A). Broadly, PCA is an unsupervised multi-variate dimensionality reduction technique that combines and reduces the input variables into new features that retain properties of the original data while maximizing the variance of the data accounted for.^60,61^ Syndromic visualization encapsulates a set of plots (e.g., syndromic plots, barmaps, and heatmaps) developed in the Ferguson lab to intuitively present the PCA results.^40,62^

MVA revealed that the aggregated data set has 1.3% missingness (Fig. 4B). Within the Chou and colleagues 2018 data, one of the samples (i.e., rows which correspond to individual mice in the selected data sets) was missing values across all six variables. A sample that is missing values across all variables cannot be accurately imputed, so we removed the sample from further analysis. Two other samples were missing values for Ym1. Harnessing experimenter knowledge, we identified that the two values are missing because of technical errors during the qPCR procedure. We further verified that the missing data could be considered MCAR with Little's statistical test (*p* = 0.86),^36^ indicating that the pattern of missingness was attributable to random chance and not correlated with the values of other variables in the data set.

Importantly, data imputation could accordingly proceed without the need to explicitly model the missingness.^58,59^ We imputed the two missing values using a multiple imputation method that operates under the assumption that the data are MCAR: predictive mean matching. The imputation first creates a predictive model from the samples with complete cases (rows without missing values) to generate estimates for the missing values, identifies which complete samples have observed values closest to the predicted value for the missing entry, and then randomly chooses one of the observed values to use for the imputation. We repeated the process 10 times—a general guideline for multiple imputation of missing data that is sufficient for cases where only a small portion (<10%) of the data is missing^63^—to create 10 imputed data sets.

We then performed PCA on each individual imputed data set with mean centering and z-scaling of the data (i.e., analogous to running PCA on the correlation matrix of the data set) to examine the relationship between the six inflammatory markers. In brief, during experimental design, researchers select outcome measures (i.e., variables) that represent broader domains of interest. In our use case, the variables are inflammatory markers that can be categorized into pro-inflammatory, anti-inflammatory, and oxidative stress domains.^33,34^ PCA transforms the variables of this multi-variate data set into a set of PCs that capture the relationship of the analytes. PCA maximizes the variance in the data along each PC under the restriction that each component is uncorrelated to the others.^60,61^ Importantly, because PCA is an unsupervised method, the PCs are data-derived scores determined purely by the correlational pattern between the variables as observed. PCA thus captures the relationship between the represented domains in a data-driven manner and can identify new underlying (i.e., latent) cross-domain features (Fig. 4C). We can further visualize the contributions (i.e., loadings) of each of the original variables to each PC in the form of a syndromic plot (Fig 4D).

The initial PCA revealed three PCs that were above the scree plot elbow with eigenvalues of 2.88 (PC1), 1.22 (PC2), and 0.91 (PC3). Examination of loadings suggested that PC3 included parts of TBI biology that are of historical interest to the field, so we opted to include it in subsequent stability/accuracy testing and discussion. To determine whether our imputation method significantly affected our PCA output, we tested the similarity of the resultant PCs from PCAs performed on each of the individual imputed data sets. We found that the resultant PCAs were almost exactly identical (congruence coefficient, >0.999 ± 0.001 for each PC; Cattell's salient similarity = 1.000 ± <0.001 for each PC). Accordingly, we took the mean of the imputed values to create a single imputed, complete data set and then applied PCA for further analysis. We additionally verified that the PCA results of the mean-imputation data set were near identical to the PCAs of each individual imputed data set (congruence coefficient, >0.999 ± 0.001 for each PC; Cattell's salient similarity = 1.000 ± 0.001 for each PC; RMSE = 0.0010 ± 0.0003 for PC1, 0.0010 ± 0.0007 for PC2, and 0.0010 ± 0.0006 for PC3).

**FIG. 5.**
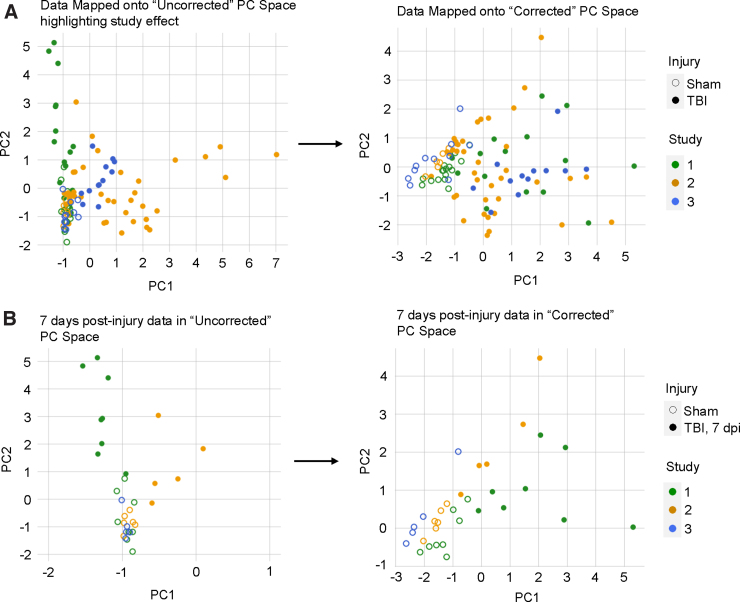
Change in PC scores after correcting for study. (**A**) Data points mapped onto PC space (i.e., PC1 and PC2) grouped by Study and Injury groups. In the uncorrected PC space, PC1 primarily captured the variance from study 2 whereas PC2 primarily captured the variance from study 1 (left). Two-way ANOVA revealed significant main effects of Study and Injury and significant interaction along both PC1 and PC2. After correcting for study, PC1 primarily captured the variance between sham and TBI samples, and neither PC1 nor PC2 appeared to represent the variance from a single study (right). Two-way ANOVA revealed only a significant main effect of Injury along PC1. (**B**) Data points for animals belonging to similar experimental groups mapped onto the uncorrected and study-corrected PC spaces. Before correcting for the study effect, adult animals at 7 days post-injury (dpi) from study 1 and study 2 fell on opposite sides of the sham experimental groups (left). After correcting for study, the 7-dpi animals clustered more closely in the PC space and exhibited similar PC1 direction in relation to sham animals (right). The variance accounted for (VAF) of the PCs additionally show that the study correction increases the VAF of PC1 and decreases the VAF of PC2. ANOVA, analysis of variance; PC, principal component; TBI, traumatic brain injury.

The resulting loadings of each variable to each PC allowed us to transform the original data into PC scores for each subject. Plotting the PC scores on the first two PC axes, we observed that the first two components account for variance attributable to study 2 and study 1 (along PC1 and PC2 respectively; Fig. 5A). A two-way ANOVA of PC1 scores revealed significant main effects of Injury (*F*_(1,93)_ = 8.30, *p* < 0.005) and Study (*F*_(2,93)_ = 18.32, *p* < 0.001) and a significant interaction of Injury and Study (*F*_(2,93)_ = 4.84, *p* < 0.05). Along PC2, two-way ANOVA similarly revealed significant main effects of Injury (*F*_(1,93)_ = 46.69, *p* < 0.001) and Study (*F*_(2,93)_ = 4.64, *p* < 0.05) and a significant interaction of Injury and Study (*F*_(2,93)_ = 5.63, *p* < 0.005). To further emphasize the study effect captured by the PC scores, we filtered for all adult sham animals and adult TBI animals at 7 dpi across the three cohorts. We observed that the TBI animals fell on both sides of the sham animals along PC1, suggesting that the PCA had transformed the original data according to variance attributable to study as well as biological differences attributable to injury (Fig. 5B).

To correct for this study effect, we standardized each inflammatory marker into z-scores of the distribution of values within the individual studies (i.e., within-study z-score standardization; Fig. 4A). After the correction, we reperformed imputation and PCA on the standardized data set. Two-way ANOVA on the new PC scores revealed only a main effect of Injury along PC1 (*F*_(1,93)_ = 84.42, *p* < 0.001). There were no significant main effects or interactions with Study for either PC1 or PC2, suggesting successful correction. Visualization of the study-corrected PC scores also showed that PC1 now primarily captured the variance attributable to injury (Fig. 5A,B), verifying that our within-study z-score standardization helped to correct for the variance between studies that may have been caused by different experimenters.

**FIG. 6.**
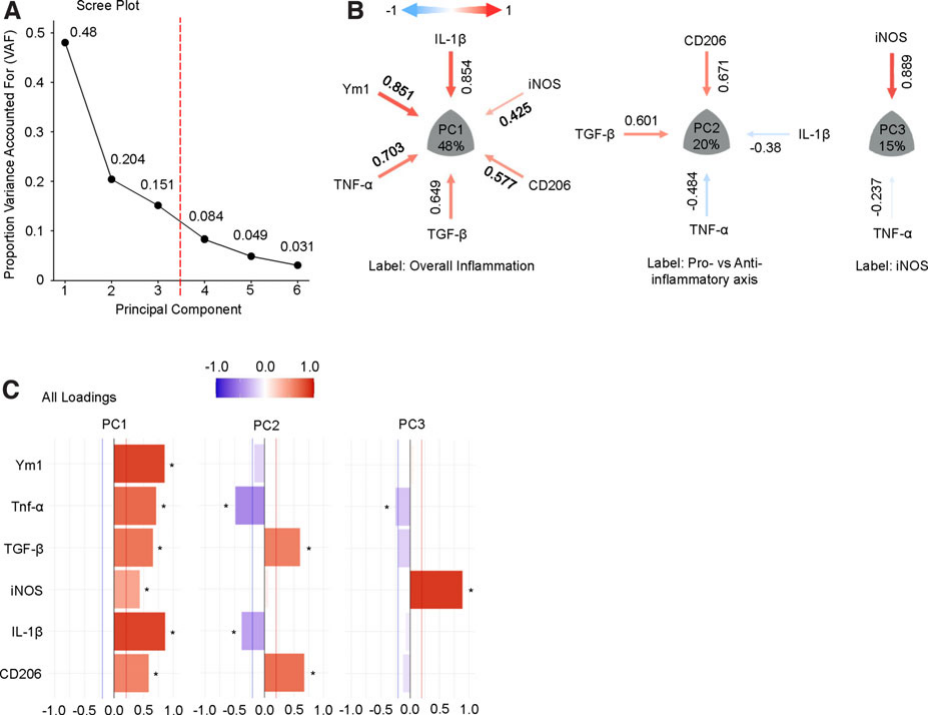
Syndromic visualization of the principal component analysis (PCA). (**A**) The scree plot after running PCA on the imputed data set revealed that the first three PCs account for 83.5% of the variance in the aggregated data set. (**B**) Syndromic plot visualization showed the significant variable loadings for each PC. PC1 was labeled as overall inflammation, PC2 as the pro- versus anti-inflammatory axis, and PC3 as iNOS expression. (**C**) The barmap visualization provides additional information, including the variable loadings that were below the threshold of significance (0.2) for each PC. The barmap denotes with an asterisk (“*”) which loadings were above the significance threshold. CD206, cluster of differentiation 206; IL-1β, interleukin 1 beta; iNOS, inducible nitric oxide synthase; PC, principal component; TGF-β, transforming growth beta; TNF-α, tumor necrosis factor alpha; Ym1, Ym1 chitinase-like protein.

Taking the PCA of the data set that had been standardized to within-study z-scores and then averaged across 10 imputations, we plotted the VAF by each PC on a scree plot. We observed that the first three PCs (PC1, PC2, and PC3) accounted for 83.5% of the variance in the aggregated data set (Fig. 6A). We focused our attention on these three PCs given that they explain the majority of the data variance and have biologically interpretable loading patterns; conversely, PC4–6 essentially captured unexplained variance and noise in the data. We visualized the loadings of each individual marker to the first three PCs using the syndRomics package in R to generate syndromic plots (Fig. 6B).^40^ Markers visualized in the syndromic plots were those with absolute loadings above a threshold of significance (|loading| > 0.2).

We also visualized the PCA output as a barmap and heatmap, which show the loadings for all six inflammatory markers to each PC (Fig. 6C and Supplementary Fig. S2). Researchers with domain expertise in pre-clinical TBI neuroinflammation examined the loading patterns and labeled PC1 as representative of an “overall inflammation” axis, with every inflammatory marker loading positively, and PC2 as representative of the “pro- vs anti-inflammatory” axis, with anti-inflammatory markers (CD206 and TGF-β) loading inversely to proinflammatory markers (IL-1β and TNF-α). Last, PC3 showed iNOS loading almost exclusively, suggesting that iNOS might provide unique information about the inflammatory state after injury distinct from the other five markers.

**FIG. 7.**
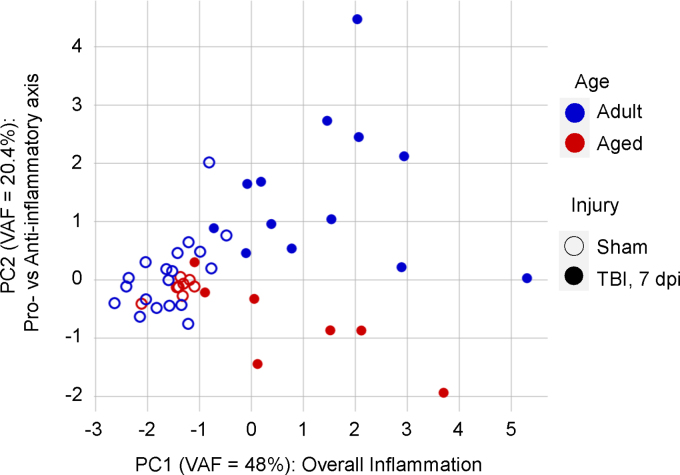
Validation of results from previous studies with the aggregate analysis. Animals corresponding to sham versus TBI at 7 days post-injury and Adult (3 months) versus Aged (18+ months) experimental groups were filtered from the aggregated data set and mapped onto the study-corrected PC space. Sham animals clustered closely regardless of age. TBI significantly increased the overall inflammation (PC1; variance accounted for [VAF] = 48%) for TBI animals without a significant main effect of Age or interaction. Along PC2 (VAF = 20.4%), there were significant effects of Injury and Age as well as a significant Injury and Age interaction, suggesting that aged animals exhibited a shift toward proinflammation whereas adult animals shifted toward anti-inflammation at 7 days post-injury. PC, principal component; TBI, traumatic brain injury.

Notably, data aggregation and PCA can increase the sensitivity (increased effect sizes) to better distinguish experimental groups as compared to univariate analyses. To illustrate this, we mapped the PC scores for adult (3–6 months) and aged (18^+^ months) animals in sham or 7-dpi experimental groups from the aggregated data set (*n* = 47; Fig. 7). We observed that TBI increased the inflammatory profile at 7 dpi as represented by an increase in PC1 score (Injury main effect: *F*_(1,43)_ = 65.14, *p* < 0.001). Further, we observed a distinct separation between adult and aged animals at 7 dpi along PC2: Aged TBI animals have lower PC2 scores as compared to adult TBI animals, reflecting an age-driven shift toward proinflammation and away from anti-inflammation at the subchronic time point (two-way ANOVA; Injury main effect: *F*_(1,43)_ = 7.44, *p* < 0.01; Age main effect: *F*_(1,43)_ = 18.69, *p* < 0.001; Injury and Age interaction: *F*_(1,43)_ = 15.02, *p* < 0.001; Tukey's HSD: adult TBI vs. aged TBI, *p* < 0.001).

For comparison, we also reproduced the univariate analyses from Chou and colleagues 2018 with the individual inflammatory markers and the study-specific cohort.^33^ We calculated the effect sizes (η^2^) and corresponding observed statistical power (1-β) for the main effects and interactions of Injury and Age for PC1, PC2, and each individual marker (Supplementary Table S1). PC1 had the largest effect size for Injury (η^2^ = 0.593) with an observed power of 1.0. Of particular interest to the original study, which examines Injury and Age interactions, PC2 had the largest effect size for the interaction term (η^2^ = 0.179) with an observed power of 0.96. Importantly, PCs are derived mathematically from correlations in the data and directly model the relationship between variables without relying on multiple univariate comparisons, which would be prone to false positives. PCA thus not only improved sensitivity for the original experimental question, but also specifically established that age skews subchronic inflammation away from anti-inflammation and toward proinflammation. This was not clearly observed in the original cohort and univariate analyses with the individual markers.

Altogether, our analyses demonstrate the potential of multi-dimensional analytics in reinforcing inferential reproducibility of previous findings while leveraging heterogeneous data sets to identify persistent pathophysiological patterns. By establishing a functional infrastructure toward the FAIR principles of data sharing to promote data reuse, the ODC-TBI acts as a critical bridge for data-set standardization and aggregation to facilitate and accelerate such efforts.

## Discussion

The ODC-TBI is a data commons developed for pre-clinical TBI research designed to 1) enable data sharing within the research community,^12^ 2) support data standardization guidelines established by the NINDS,^20,21^ 3) promote FAIR data-sharing principles,^25^ and 4) empower Big Data analytics in pre-clinical TBI research.^10^ Importantly, the ODC-TBI is uniquely positioned to directly socialize and implement data sharing with the TBI research community. In contrast to general cross-disciplinary repositories that have few requirements on structure and documentation, the ODC-TBI requires data sets to be organized in the Tidy data format as well as include critical information through data dictionaries and data-set–associated metadata. Such standards significantly improve data interoperability and reusability and reduce the likelihood of shared data being mis- or uninterpretable for reuse.

Further, because the ODC-TBI is developed in close communication with the TBI research community, we are able to directly assist and empower researchers to understand and meet the FAIR data standards as well as continue to evolve the ODC-TBI platform to meet community needs. This critical community engagement sets the ODC-TBI apart from all-purpose repositories and follows a similar path to success for FAIR data sharing as demonstrated by other community-centered data repositories.^64^ We additionally demonstrate an analytical process made possible by utilizing the ODC-TBI to identify common TBI immune responses across three different pre-clinical TBI studies. Although the analysis performed here is not yet integrated on the ODC-TBI, analytical features and tools are being actively developed that will be directly available through the platform. Indeed, as the ODC-TBI continues to grow to fulfill the needs and interests of the TBI research community, the platform will evolve to be both a data repository as well as a hypothesis generation platform that empowers researchers to leverage the richness of individual subject-level data through FAIR data sharing and publication.

We recognize that the practice of data sharing is still emerging in many biological fields, including pre-clinical TBI. There are potential risks of data sharing—such as data security and misuse without proper citation^45,46^—that endure among research communities even as publishers and funding agencies have begun to require it.^23,47,65^ With the ODC-TBI, we ensure that the PI has full control of the accessibility of their data set when sharing their work with their peers in the research community and when they publish the data sets to the general public. The ODC-TBI also tracks which users have accessed shared data sets and provides the information to the data-set PIs. During data-set publication, the ODC-TBI generates a unique and persistent DOI and citation for the data set where the PI can include all associated authors and contributors for proper credit.

Importantly, all data sets published through the ODC-TBI are done so under the Creative Commons CC-BY 4.0 license, meaning that any work utilizing those data sets must properly cite them much the same way scientific articles are cited. This provides a novel avenue for researchers to benefit from their data as a new, citable, scientific work product. This has direct benefits to the data contributor, given that data sharing has been found to be associated with an increase in citations for researchers.^66^ These requirements are explicitly written as part of the data-use agreement consented to by all users signing up to the ODC-TBI and provide a key layer of accountability. Future features of the ODC-TBI platform will include direct peer-to-peer sharing functionalities, further diversifying the methods that PIs can upload and share their data in a protected manner on the ODC-TBI.

We also realize the importance of supporting and integrating common terminology, such as CDEs, to improve all aspects of FAIR data sharing on the ODC-TBI. Indeed, NINDS and the TBI research community have recognized the challenges in data comparison attributable to the lack of common variable names and definitions; this spurred a concerted multi-center endeavor to identify and define CDEs to be adopted by clinical and pre-clinical TBI researchers.^15,20,21^ We manually aligned variables in 11 data sets described here to NINDS-defined CDEs before uploading them to the ODC-TBI.

To promote the practice of aligning NINDS-defined CDEs, we aim to implement a CDE mapping system on the ODC-TBI built upon the engineering framework of the InterLex/NeuroLex system developed by NIF.^22^ The feature will enable CDE mapping after data upload and allow users to align each variable of a data set to a dictionary of CDEs (including NINDS-defined CDEs), an aspect of the ODC-TBI that further distinguishes the repository from other existing platforms. The mapping system would increase the accessibility and prominence of NINDS-defined CDEs and help to construct a knowledge base of TBI research to make data more findable, interoperable, and reusable. As the number of data sets shared on the ODC-TBI grows, it will be possible to further validate the prevalence of NINDS-defined CDEs as well as identify novel CDEs in TBI research.

We expect many data sets uploaded to the ODC-TBI to contain missing values for a variety of reasons, such as those visualized in Figure 3. MVA is a critical component for Big Data analytics; many multi-variate techniques as well as common univariate approaches (*t*-test, correlation, and ANOVA) require complete data sets for analysis. Most commercial statistics tools default to dropping subjects (list-wise deletion) with missing values. However, researchers are often unaware of the impact of missing values, and the practice of list-wise deletion can introduce bias and contribute to scientific irreproducibility.^36,58^ Understanding the types of missingness is essential for selecting which data imputation technique can be applied; various imputation techniques contain different assumptions that would invalidate specific analyses if they are violated.^58,67^ In simple cases, such as when the Treatment column is not applicable in the study but still kept as a column (red-labeled cells in Fig. 3), the missing value can be imputed with a control value (e.g., control, naïve, or zero).

More generally, data imputation depends on modeling the correlation between variables, and if two variables are never collected in tandem because of experimental design or limitations, then identifying their relationship becomes increasingly inaccurate. Recognizing when data are missing because of experimental design is critical. To this end, the ODC-TBI supports the upload of data-set–associated methodology and data dictionaries that can provide context for researchers to interpret when data imputation is appropriate.

Similarly, the methodology documents and data dictionaries can also highlight the reasons data may be missing because of technical reasons. In many cases, data are missing because of truly random events (e.g., contamination of a single sample during processing or human error performing the experimental protocol). In such cases, the missing data would be classified as MCAR, which permits straightforward approaches to imputing missing data without having to incorporate patterns of missingness directly into the analysis.^58^ In other circumstances, the data might be MNAR: The missingness is correlated to one of the other variables of interest or itself. For example, this may occur when a sample's protein quantification falls below the detectable range of an assay. Instead of keeping a potentially inaccurate value, the experimenter decides to exclude the value altogether.

Here, the missingness is correlated to the variable itself: The value is missing because its value falls below the detectable limit. If we were to impute the missing values without regard, we would overestimate the true values of the missing data and bias our analyses. With proper documentation, the type of data missingness can be identified to better inform the appropriate approach to data imputation and avoid grave statistical mistakes in analysis.

As an illustration of how ODC-TBI data can be reused for further discovery, we pooled data across three cohorts of subjects from past articles^33,34^ and performed a multi-variate analysis workflow. Our results indicate that across experiments investigating the effects of time post-injury, age, and a monocyte-infiltration antagonist, there were latent variables (i.e., PCs) that captured the general inflammatory state (PC1), a pro- vs anti-inflammatory state (PC2), and oxidative stress response (PC3). PC loadings further suggest that: 1) Ym1 is not a primary contributor to explaining the pro- versus anti-inflammatory state of the tissue despite Ym1 being considered an anti-inflammatory marker on myeloid cells^68^; 2) whereas myeloid cells do not exhibit strictly pro- or anti-inflammatory phenotypes after TBI, there is a marked inverse relationship between pro- and anti-inflammatory markers^34,50^; and 3) iNOS expression is mostly distinct from the other analytes even though iNOS is often correlated with proinflammatory responses in innate immune cells after TBI.^69,70^ This is further reinforced by the fact that PC3—which is where iNOS primarily loads into—has an eigenvalue of 0.91; a PC with an eigenvalue of <1 would mean that the PC contains less variance or information than any individual marker.

We chose to report PC3 given that the eigenvalue is close to 1, the component is clearly biologically interpretable, and that the inference regarding iNOS is consistent with or without retaining PC3. Applications of PCA onto more complex data that lack clear interpretability should also consider utilizing other PC selection methods, such as the Kaiser rule, based on eigenvalues or permutation tests. Additionally, though we focused on interpreting the PCs as composite biological responses across the variety of experimental conditions, principal component regression (PCR) could be used in future studies to identify the PCs with maximal prediction accuracy for a given hypothesis. Depending on the hypothesis of interest, PCR may reveal that lower variance PCs may, in fact, be better predictors and thus offer additional insight to the relationship of the PCs to specific biological questions.

Overall, these results reinforce the inferential reproducibility of previous findings through the increased sample size from data aggregation and the methodology that specifically reveals persistent latent variables across different experimental manipulations. Additionally, as more data sets are shared, such analyses can similarly uncover the multi-variate correlation of other measured variables, such as from behavior or imaging assays. This can be further applied across outcome domains to generate novel hypotheses about latent biological responses after TBI, which, in turn, can stimulate the conceptualization and design of new experiments.

Notably, in the original univariate analysis of Chou and colleagues (2018), we observed a general impairment of anti-inflammatory markers, but no difference in proinflammatory markers at 7 dpi in aged animals after TBI.^33^ Although the univariate analysis suggested a shift in pro- versus anti-inflammatory responses, at best we could conclude that anti-inflammation after TBI was blunted by age. As reflected in our analysis by PC2, we can, in fact, posit that age biases the subchronic inflammatory state toward proinflammation and away from anti-inflammation. Further, the effect-size analysis shows that data pooling and PCA increased the overall power for detecting the primary experimental effect of interest (i.e., interaction of age and injury). Indeed, uni- and multi-variate analyses together provide a more meaningful understanding of the biology, with the latter additionally increasing the statistical power and inferential validity of findings.

Critically, the work demonstrated here is limited by the amount of data on the ODC-TBI that can be effectively aggregated. In particular, the analysis was significantly facilitated by first-hand familiarity with the data, though proper documentation of the data sets as described will go a long way to reinforce data interpretability for reuse. However, there remains a considerable obstacle of the heterogeneity of outcome variables in the field. Indeed, whereas the aggregated data originated from three separate experimental studies, they ultimately comprised a total sample size of 99 subjects—larger than typical individual animal experiments, but still comparatively small—out of our total data. Importantly, these 99 subjects were the samples with data across the six analytes.

Fortunately, whereas many multi-variate techniques rely on subjects having complete data for the same variables, Big Data methods for handling data variety may provide avenues for extracting novel inferences and hypotheses from data sets that are not completely overlapping with variables. For example, validated latent variables may provide common multi-variate domains to connect data sets with only partially overlapping variables. Additionally, there is significant work on extending concepts of missing data imputation toward incomplete multi-table data sets that could be applied toward these challenges.^71^ As the community use of ODC-TBI for data sharing and reuse grows, understanding the differences in collected data may also provide an impetus for standardizing outcome variables for common biological and functional pathways.

Notably, the analytical workflow presented here can be extended to reveal features persistent across laboratories, experimenters, injury parameters, and injury models. This approach is powerful because it ultimately leverages the heterogeneity of experimental design in pre-clinical TBI research to find common underlying pathophysiology of TBI. Critically, although the PCs are extracted through multi-variate statistical techniques, assigning labels and contextualizing PCs with the underlying biology requires a combination of statistical rules based in the well-established field of factor analysis^72,73^ as well as the specific biomedical domain expertise. Data sharing through the ODC-TBI will open avenues of collaboration not only between researchers in pre-clinical TBI, but also between computational and molecular researchers who can provide complementary expertise toward interpreting results. As more studies populate the ODC-TBI, such opportunities and interdisciplinary collaboration will identify features of TBI across an even broader array of heterogeneity and uncover possible therapeutic targets and biomarkers that would be applicable to a broader patient population.

### Data availability

The data sets generated and analyzed in the current study will be published and made publicly available in the ODC-TBI repository. The datasets generated and analyzed in the datasets can be identified by their DOIs: 10.34945/F51P49 (Fig. 2), 10.34945/F5T595 (Fig. 3–7), and 10.34945/F5PC77 (Figs. 4–7).

## Supplementary Material

Supplemental data

Supplemental data

Supplemental data

Supplemental data

## Supplementary Material

Supplementary R Code

## Abbreviations Used

ANOVA: analysis of variance
CD206: cluster of differentiation 206
CDEs: Common Data Elements
DOI: digital object identifier
dpi: day(s) post-injury
FAIR: Findable, Accessible, Interoperable, and Reusable
HSD: honestly significant difference
IL-1β: interleukin 1-beta
iNOS: inducible nitric oxide synthase
MAR: missing at random
MCAR: missing completely at random
MNAR: missing not at random
MVA: missing values analysis
NIF: Neuroscience Information Framework
NINDS: National Institute of Neurological Disorders and Stroke
PCs: principal components
PCA: principal component analysis
PI: Principal Investigator
PCR: principal component regression
qPCR: quantitative polymerase chain reaction
RMSE: root mean square error
TBI: traumatic brain injury
TGF-β: transforming growth factor beta
TNF-α: tumor necrosis factor alpha
UCSF: University of California San Francisco
VAF: variance accounted for
Ym1: *Ym1* chitinase-like protein

## References

[1] Centers for Disease Control and Prevention. (2015). Traumatic Brain Injury In the United States: Epidemiology and Rehabilitation. National Center for Injury Prevention and Control; Division of Unintentional Injury Prevention: Atlanta, GA.

[2] Dewan, M.C., Rattani, A., Gupta, S., Baticulon, R.E., Hung, Y.-C., Punchak, M., Agrawal, A., Adeleye, A.O., Shrime, M.G., Rubiano, A.M., Rosenfeld, J.V., and Park, K.B. (2018). Estimating the global incidence of traumatic brain injury. J. Neurosurg. 130, 1080–1097. 10.3171/2017.10.JNS17352 29701556

[3] Zaloshnja, E., Miller, T., Langlois, J.A., and Selassie, A.W. (2008). Prevalence of long-term disability from traumatic brain injury in the civilian population of the United States, 2005. J. Head Trauma Rehabil. 23, 394–400. 19033832 10.1097/01.HTR.0000341435.52004.ac

[4] Masel, B.E., and DeWitt, D.S. (2010). Traumatic brain injury: a disease process, not an event. J. Neurotrauma 27, 1529–1540. 20504161 10.1089/neu.2010.1358

[5] Xiong, Y., Mahmood, A., and Chopp, M. (2009). Emerging treatments for traumatic brain injury. Expert Opin. Emerg. Drugs 14, 67–84. 19249984 10.1517/14728210902769601 PMC2773142

[6] Maas, A.I.R., Roozenbeek, B., and Manley, G.T. (2010). Clinical trials in traumatic brain injury: past experience and current developments. Neurotherapeutics 7, 115–126. 20129503 10.1016/j.nurt.2009.10.022 PMC5084118

[7] Saatman, K.E., Duhaime, A.-C., Bullock, R., Maas, A.I.R., Valadka, A., and Manley, G.T. (2008). Classification of traumatic brain injury for targeted therapies. J. Neurotrauma 25, 719–738. 18627252 10.1089/neu.2008.0586 PMC2721779

[8] Ng, S.Y., and Lee, A.Y.W. (2019). Traumatic brain injuries: pathophysiology and potential therapeutic targets. Front. Cell. Neurosci. 13, 528. 31827423 10.3389/fncel.2019.00528 PMC6890857

[9] Xiong, Y., Mahmood, A., and Chopp, M. (2013). Animal models of traumatic brain injury. Nat. Rev. Neurosci. 14, 128–142. 23329160 10.1038/nrn3407 PMC3951995

[10] Huie, J.R., Almeida, C.A., and Ferguson, A.R. (2018). Neurotrauma as a big-data problem: Curr. Opin. Neurol. 31, 702–708. 10.1097/WCO.0000000000000614 PMC7075373 30379703

[11] Agoston, D.V., and Langford, D. (2017). Big Data in traumatic brain injury; promise and challenges. Concussion 2, CNC44. 10.2217/cnc-2016-0013 PMC6122694 30202589

[12] Hawkins, B.E., Huie, J.R., Almeida, C., Chen, J., and Ferguson, A.R. (2019). Data dissemination: shortening the long tail of traumatic brain injury dark data. J. Neurotrauma 37, 2414–2423. 30794049 10.1089/neu.2018.6192 PMC7698976

[13] Buonora, J.E., Yarnell, A.M., Lazarus, R.C., Mousseau, M., Latour, L.L., Rizoli, S.B., Baker, A.J., Rhind, S.G., Diaz-Arrastia, R., and Mueller, G.P. (2015). Multivariate analysis of traumatic brain injury: development of an assessment score. Front. Neurol. 6, 68. 25870583 10.3389/fneur.2015.00068 PMC4378282

[14] Huie, J.R., Diaz-Arrastia, R., Yue, J.K., Sorani, M.D., Puccio, A.M., Okonkwo, D.O., Manley, G.T., Ferguson, A.R., the TRACK-TBI Investigators, Adeoye, O.M., Badjatia, N., Boase, K.D., Bodien-Guller, Y., Bullock, M.R., Chesnut, R.M., Corrigan, J.D., Crawford, K.L., Diaz-Arrastia, R., Dikmen, S.S., Duhaime, A.-C., Ellenbogen, R.G., Ezekiel, F., Feeser, V.R., Giacino, J.T., Goldman, D.P., Gonzales, L., Gopinath, S.P., Gullapalli, R.P., Hemphill, J.C., Hotz, G.A., Kramer, J.H., Levin, H., Lindsell, C.J., Machamer, J., Madden, C., Markowitz, A.J., Martin, A., Mathern, B.E., McAllister, T.W., McCrea, M.A., Merchant, R.E., Noel, F., Perl, D.P., Puccio, A.M., Rabinowitz, M., Robertson, C.S., Rosand, J., Sander, A.M., Satris, G., Schnyer, D.M., Seabury, S.A., Sergot, P., Sherer, M., Stein, D.M., Stein, M.B., Taylor, S.R., Temkin, N.R., Toga, A.W., Turtzo, L.C., Vespa, P.M., Wang, K.K., Zafonte, R., and Zhang, Z. (2019). Testing a multivariate proteomic panel for traumatic brain injury biomarker discovery: a TRACK-TBI Pilot Study. J. Neurotrauma 36, 100–110. 30084741 10.1089/neu.2017.5449 PMC6306686

[15] Thompson, H.J., Vavilala, M.S., and Rivara, F.P. (2015). Chapter 1 Common Data Elements and federal interagency traumatic brain injury research informatics system for TBI research. Annu. Rev. Nurs. Res. 33, 1–11. 25946381 10.1891/0739-6686.33.1 PMC4704986

[16] Nielson, J.L., Paquette, J., Liu, A.W., Guandique, C.F., Tovar, C.A., Inoue, T., Irvine, K.-A., Gensel, J.C., Kloke, J., Petrossian, T.C., Lum, P.Y., Carlsson, G.E., Manley, G.T., Young, W., Beattie, M.S., Bresnahan, J.C., and Ferguson, A.R. (2015). Topological data analysis for discovery in preclinical spinal cord injury and traumatic brain injury. Nat. Commun. 6, 8581. 26466022 10.1038/ncomms9581 PMC4634208

[17] Yue, J.K., Vassar, M.J., Lingsma, H.F., Cooper, S.R., Okonkwo, D.O., Valadka, A.B., Gordon, W.A., Maas, A.I.R., Mukherjee, P., Yuh, E.L., Puccio, A.M., Schnyer, D.M., and Manley, G.T.; TRACK-TBI Investigators. (2013). Transforming research and clinical knowledge in traumatic brain injury pilot: multicenter implementation of the Common Data Elements for traumatic brain injury. J. Neurotrauma 30, 1831–1844. 23815563 10.1089/neu.2013.2970 PMC3814815

[18] Steyerberg, E.W., Wiegers, E., Sewalt, C., Buki, A., Citerio, G., Keyser, V.D., Ercole, A., Kunzmann, K., Lanyon, L., Lecky, F., Lingsma, H., Manley, G., Nelson, D., Peul, W., Stocchetti, N., Steinbüchel, N. von, Vyvere, T.V., Verheyden, J., Wilson, L., Maas, A.I.R., and Menon, D.K.; CENTER-TBI Participants and Investigators. (2019). Case-mix, care pathways, and outcomes in patients with traumatic brain injury in CENTER-TBI: a European prospective, multicentre, longitudinal, cohort study. Lancet Neurol. 18, 923–934. 31526754 10.1016/S1474-4422(19)30232-7

[19] Ferguson, A.R., Nielson, J.L., Cragin, M.H., Bandrowski, A.E., and Martone, M.E. (2014). Big data from small data: data-sharing in the “long tail” of neuroscience. Nat. Neurosci. 17, 1442–1447. 25349910 10.1038/nn.3838 PMC4728080

[20] Smith, D.H., Hicks, R.R., Johnson, V.E., Bergstrom, D.A., Cummings, D.M., Noble, L.J., Hovda, D., Whalen, M., Ahlers, S.T., LaPlaca, M., Tortella, F.C., Duhaime, A.-C., and Dixon, C.E. (2015). Pre-clinical traumatic brain injury Common Data Elements: toward a common language across laboratories. J. Neurotrauma 32, 1725–1735. 26058402 10.1089/neu.2014.3861 PMC4651035

[21] LaPlaca, M.C., Huie, J.R., Alam, H.B., Bachstetter, A.D., Bayir, H., Bellgowan, P.S.F., Cummings, D., Dixon, C.E., Ferguson, A.R., Ferland-Beckham, C., Floyd, C., Friess, S., Galanopoulou, A., Hall, E.D., Harris, N.G., Hawkins, B.E., Hicks, R., Hulbert, L.E., Johnson, V.E., Kabitzke, P., Lafrenaye, A.D., Lemmon, V., Lifshitz, C., Lifshitz, J., Loane, D.J., Misquitta, L., Nikolian, V.C., Noble, L., Smith, D.H., Taylor-Burds, C., Umoh, N., Vovk, O., Williams, A.M., Young, M., and Zai, L. (2021). Preclinical Common Data Elements for traumatic brain injury research: progress and use cases. J. Neurotrauma 38, 1399–1410. 33297844 10.1089/neu.2020.7328 PMC8082734

[22] Larson, S.D., and Martone, M.E. (2013). NeuroLex.org: an online framework for neuroscience knowledge. Front. Neuroinformatics 7, 18. 10.3389/fninf.2013.00018 PMC3757470 24009581

[23] Callahan, A., Anderson, K.D., Beattie, M.S., Bixby, J.L., Ferguson, A.R., Fouad, K., Jakeman, L.B., Nielson, J.L., Popovich, P.G., Schwab, J.M., and Lemmon, V.P. (2017). Developing a data sharing community for spinal cord injury research. Exp. Neurol. 295, 135–143. 28576567 10.1016/j.expneurol.2017.05.012 PMC6448396

[24] Fouad, K., Bixby, J.L., Callahan, A., Grethe, J.S., Jakeman, L.B., Lemmon, V.P., Magnuson, D.S.K., Martone, M.E., Nielson, J.L., Schwab, J.M., Taylor-Burds, C., Tetzlaff, W., Torres-Espin, A., Ferguson, A.R., the FAIR-SCI Ahead Workshop Participants, Alam, S., Bacon, M., Bambrick, L., Basso, M., Beattie, M., Bresnahan, J., Gensel, J., Graham, D., Grethe, J., Russell Huie, J., Jones, L., Kabitzke, P., Kleitman, N., Kusiak, A., Kwon, B., Lederer, R., MacLeod, M., May, V., Neff, E., and Rabchevsky, S. (2020). FAIR SCI ahead: the evolution of the Open Data Commons for pre-clinical spinal cord injury research. J. Neurotrauma 37, 831–838. 31608767 10.1089/neu.2019.6674 PMC7071068

[25] Wilkinson, M.D., Dumontier, M., Aalbersberg, Ij.J., Appleton, G., Axton, M., Baak, A., Blomberg, N., Boiten, J.-W., da Silva Santos, L.B., Bourne, P.E., Bouwman, J., Brookes, A.J., Clark, T., Crosas, M., Dillo, I., Dumon, O., Edmunds, S., Evelo, C.T., Finkers, R., Gonzalez-Beltran, A., Gray, A.J.G., Groth, P., Goble, C., Grethe, J.S., Heringa, J., 't Hoen, P.A.C., Hooft, R., Kuhn, T., Kok, R., Kok, J., Lusher, S.J., Martone, M.E., Mons, A., Packer, A.L., Persson, B., Rocca-Serra, P., Roos, M., van Schaik, R., Sansone, S.-A., Schultes, E., Sengstag, T., Slater, T., Strawn, G., Swertz, M.A., Thompson, M., van der Lei, J., van Mulligen, E., Velterop, J., Waagmeester, A., Wittenburg, P., Wolstencroft, K., Zhao, J., and Mons, B. (2016). The FAIR Guiding Principles for scientific data management and stewardship. Sci. Data 3, 160018. 26978244 10.1038/sdata.2016.18 PMC4792175

[26] Wickham, H. (2014). Tidy data. J. Stat. Softw. 59, 1–23.26917999

[27] R Core Team. (2020). R: A Language and Environment for Statistical Computing. R Foundation for Statistical Computing: Vienna, Austria.

[28] Wickham, H., Averick, M., Bryan, J., Chang, W., McGowan, L., François, R., Grolemund, G., Hayes, A., Henry, L., Hester, J., Kuhn, M., Pedersen, T., Miller, E., Bache, S., Müller, K., Ooms, J., Robinson, D., Seidel, D., Spinu, V., Takahashi, K., Vaughan, D., Wilke, C., Woo, K., and Yutani, H. (2019). Welcome to the {tidyverse}. J. Open Source Softw. 4, 1686.

[29] Wickham, H. (2016). ggplot2: Elegant Graphics for Data Analysis, 2nd ed. Springer: New York.

[30] Neuwirth, E. (2014). RColorBrewer: ColorBrewer Palettes. R package version 1.1-2. https://CRAN.R-project.org/package=RColorBrewer

[31] Keitt, T. (2012). colorRamps: Builds color tables. R package version 2.3. https://CRAN.R-project.org/package=colorRamps

[32] Tierney, N., and Cook, D. (2018). Expanding tidy data principles to facilitate missing data exploration, visualization and assessment of imputations. Monash Econometrics and Business Statistics Working Papers 14/18, Monash University, Department of Econometrics and Business Statistics: Victoria, Australia.

[33] Chou, A., Krukowski, K., Morganti, J.M., Riparip, L.-K., and Rosi, S. (2018). Persistent infiltration and impaired response of peripherally-derived monocytes after traumatic brain injury in the aged brain. Int. J. Mol. Sci. 19, 1616. 10.3390/ijms19061616 PMC6032263 29848996

[34] Morganti, J.M., Jopson, T.D., Liu, S., Riparip, L.-K., Guandique, C.K., Gupta, N., Ferguson, A.R., and Rosi, S. (2015). CCR2 antagonism alters brain macrophage polarization and ameliorates cognitive dysfunction induced by traumatic brain injury. J. Neurosci. 35, 748–760. 25589768 10.1523/JNEUROSCI.2405-14.2015 PMC4293420

[35] Beaujean, A.A. (2012). BaylorEdPsych: R Package for Baylor University Educational Psychology Quantitative Courses. R package version 0.5. https://CRAN.R-project.org/package=BaylorEdPsych

[36] Little, R.J.A. (1988). A test of missing completely at random for multivariate data with missing values. J. Am. Stat. Assoc. 83, 1198–1202.

[37] van Buuren, S. and Groothuis-Oudshoorn, K. (2011). mice: Multivariate Imputation by Chained Equations in R. J. Stat. Softw. 45, 1–67.

[38] Mardia, K.V., Kent, J.T., and Bibby, J.M. (1979). Multivariate Analysis. Academic: London; New York.

[39] Venables, W.N., and Ripley, B.D. (2002). Modern Applied Statistics with S, 4th ed. Springer-Verlag: New York.

[40] Torres Espín, A., Chou, A., Huie, R., Kyritsis, N., Upadhyayula, P.S., and Ferguson, A. (2021). Reproducible analysis of disease space via principal components using the novel R package syndRomics. eLife 10, e61812. 33443012 10.7554/eLife.61812 PMC7857733

[41] Faul, F., Erdfelder, E., Buchner, A., and Lang, A.-G. (2009). Statistical power analyses using G*Power 3.1: tests for correlation and regression analyses. Behav. Res. Methods 41, 1149–1160. 19897823 10.3758/BRM.41.4.1149

[42] Champely, S. (2020). pwr: Basic Functions for Power Analysis. R package version 1.3-0. https://CRAN.R-project.org/package=pwr

[43] Meeuws, S., Yue, J.K., Huijben, J.A., Nair, N., Lingsma, H.F., Bell, M.J., Manley, G.T., and Maas, A.I.R. (2020). Common Data Elements: critical assessment of harmonization between current multi-center traumatic brain injury studies. J. Neurotrauma 37, 1283–1290. 32000562 10.1089/neu.2019.6867 PMC7249452

[44] Broman, K.W., and Woo, K.H. (2018). Data organization in spreadsheets. Am. Stat. 72, 2–10.

[45] Zinner, D.E., Pham-Kanter, G., and Campbell, E.G. (2016). The changing nature of scientific sharing and withholding in academic life sciences research: trends from national surveys in 2000 and 2013. Acad. Med. 91, 433–440. 26675188 10.1097/ACM.0000000000001028 PMC4767624

[46] Stuart, D., Baynes, G., Hrynaszkiewicz, I., Allin, K., Penny, D., Lucraft, M., and Astell, M. (2018). Whitepaper: practical challenges for researchers in data sharing.

[47] Tenopir, C., Allard, S., Douglass, K., Aydinoglu, A.U., Wu, L., Read, E., Manoff, M., and Frame, M. (2011). Data sharing by scientists: practices and perceptions. PLoS One 6, e21101. 21738610 10.1371/journal.pone.0021101 PMC3126798

[48] Chou, A., Morganti, J.M., and Rosi, S. (2016). Frontal lobe contusion in mice chronically impairs prefrontal-dependent behavior. PLoS One 11, e0151418. 26964036 10.1371/journal.pone.0151418 PMC4786257

[49] Chou, A., Krukowski, K., Jopson, T., Zhu, P.J., Costa-Mattioli, M., Walter, P., and Rosi, S. (2017). Inhibition of the integrated stress response reverses cognitive deficits after traumatic brain injury. Proc. Natl. Acad. Sci. U. S. A. 114, E6420–E6426. 28696288 10.1073/pnas.1707661114 PMC5547647

[50] Morganti, J.M., Riparip, L.-K., and Rosi, S. (2016). Call off the dog(ma): M1/M2 polarization is concurrent following traumatic brain injury. PLoS One 11, e0148001. 26808663 10.1371/journal.pone.0148001 PMC4726527

[51] Morganti, J.M., Riparip, L.-K., Chou, A., Liu, S., Gupta, N., and Rosi, S. (2016). Age exacerbates the CCR2/5-mediated neuroinflammatory response to traumatic brain injury. J. Neuroinflammation 13, 80. 27090212 10.1186/s12974-016-0547-1 PMC4835854

[52] Krukowski, K., Chou, A., Feng, X., Tiret, B., Paladini, M.-S., Riparip, L.-K., Chaumeil, M., Lemere, C., and Rosi, S. (2018). Traumatic brain injury in aged mice induces chronic microglia activation, synapse loss, and complement-dependent memory deficits. Int. J. Mol. Sci. 19, 3753. 10.3390/ijms19123753 PMC6321529 30486287

[53] Guglielmetti, C., Chou, A., Krukowski, K., Najac, C., Feng, X., Riparip, L.-K., Rosi, S., and Chaumeil, M.M. (2017). In vivo metabolic imaging of Traumatic Brain Injury. Sci. Rep. 7, 17525. 29235509 10.1038/s41598-017-17758-4 PMC5727520

[54] Nolan, A., Hennessy, E., Krukowski, K., Guglielmetti, C., Chaumeil, M.M., Sohal, V.S., and Rosi, S. (2018). Repeated mild head injury leads to wide-ranging deficits in higher-order cognitive functions associated with the prefrontal cortex. J. Neurotrauma 35, 2425–2434. 29732949 10.1089/neu.2018.5731 PMC6196749

[55] Delbary-Gossart, S., Lee, S., Baroni, M., Lamarche, I., Arnone, M., Canolle, B., Lin, A., Sacramento, J., Salegio, E.A., Castel, M.-N., Delesque-Touchard, N., Alam, A., Laboudie, P., Ferzaz, B., Savi, P., Herbert, J.-M., Manley, G.T., Ferguson, A.R., Bresnahan, J.C., Bono, F., and Beattie, M.S. (2016). A novel inhibitor of p75-neurotrophin receptor improves functional outcomes in two models of traumatic brain injury. Brain 139, 1762–1782. 27084575 10.1093/brain/aww074 PMC4892754

[56] Lee, S., Mattingly, A., Lin, A., Sacramento, J., Mannent, L., Castel, M.-N., Canolle, B., Delbary-Gossart, S., Ferzaz, B., Morganti, J.M., Rosi, S., Ferguson, A.R., Manley, G.T., Bresnahan, J.C., and Beattie, M.S. (2016). A novel antagonist of p75NTR reduces peripheral expansion and CNS trafficking of pro-inflammatory monocytes and spares function after traumatic brain injury. J. Neuroinflammation 13, 88. 27102880 10.1186/s12974-016-0544-4 PMC4840857

[57] Andersen, C.R., Wolf, J.T., Jennings, K., Prough, D.S., and Hawkins, B.E. (2020). Moody Project Survival Analysis Morris Water Maze Dataset. ODC-TBI:408. 10.34945/F51591.

[58] Nielson, J.L., Cooper, S.R., Seabury, S.A., Luciani, D., Fabio, A., Temkin, N.R., and Ferguson, A.R.; the TRACK-TBI Investigators. (2021). Statistical guidelines for handling missing data in traumatic brain injury clinical research. J. Neurotrauma 38, 2530–2537. 32008424 10.1089/neu.2019.6702 PMC8403177

[59] Little, R.J.A., and Rubin, D.B. (2019). Statistical Analysis with Missing Data, 3rd ed. Wiley: New York.

[60] Hotelling, H. (1933). Analysis of a complex of statistical variables into principal components. J. Educ. Psychol. 24, 417–441.

[61] Jolliffe, I.T., and Cadima, J. (2016). Principal component analysis: a review and recent developments. Philos. Transact. A Math. Phys. Eng. Sci. 374. 10.1098/rsta.2015.0202 PMC4792409 26953178

[62] Ferguson, A.R., Irvine, K.-A., Gensel, J.C., Nielson, J.L., Lin, A., Ly, J., Segal, M.R., Ratan, R.R., Bresnahan, J.C., and Beattie, M.S. (2013). Derivation of multivariate syndromic outcome metrics for consistent testing across multiple models of cervical spinal cord injury in rats. PLoS One 8, e59712. 23544088 10.1371/journal.pone.0059712 PMC3609747

[63] Graham, J.W., Olchowski, A.E., and Gilreath, T.D. (2007). How many imputations are really needed? Some practical clarifications of multiple imputation theory. Prev. Sci. 8, 206–213. 17549635 10.1007/s11121-007-0070-9

[64] Torres-Espín, A., Almeida, C.A., Chou, A., Huie, J.R., Chiu, M., Vavrek, R., Sacramento, J., Orr, M.B., Gensel, J.C., Grethe, J.S., Martone, M.E., Fouad, K., and Ferguson, A.R.; STREET-FAIR Workshop Participants. (2021). Promoting FAIR Data Through Community-driven Agile Design: the Open Data Commons for Spinal Cord Injury (ODC-SCI.org). Neuroinformatics. doi.org/10.1007/s12021-021-09533-8. PMC9537193 34347243

[65] Office of The Director, National Institutes of Health. (2020). NOT-OD-21-013: Final NIH Policy for Data Management and Sharing. https://grants.nih.gov/grants/guide/notice-files/NOT-OD-21-013.html. (Last accessed February 24, 2022).

[66] Piwowar, H.A., Day, R.S., and Fridsma, D.B. (2007). Sharing detailed research data is associated with increased citation rate. PLoS One 2, e308. 17375194 10.1371/journal.pone.0000308 PMC1817752

[67] Dong, Y., and Peng, C.-Y.J. (2013). Principled missing data methods for researchers. SpringerPlus 2, 222. 23853744 10.1186/2193-1801-2-222 PMC3701793

[68] Kumar, A., Alvarez-Croda, D.-M., Stoica, B.A., Faden, A.I., and Loane, D.J. (2016). Microglial/macrophage polarization dynamics following traumatic brain injury. J. Neurotrauma 33, 1732–1750. 26486881 10.1089/neu.2015.4268 PMC5065034

[69] Stoica, B.A., Loane, D.J., Zhao, Z., Kabadi, S.V., Hanscom, M., Byrnes, K.R., and Faden, A.I. (2014). PARP-1 inhibition attenuates neuronal loss, microglia activation and neurological deficits after traumatic brain injury. J. Neurotrauma 31, 758–772. 24476502 10.1089/neu.2013.3194 PMC3967421

[70] Kochanek, P.M., Jackson, T.C., Ferguson, N.M., Carlson, S.W., Simon, D.W., Brockman, E.C., Ji, J., Bayir, H., Poloyac, S.M., Wagner, A.K., Kline, A.E., Empey, P.E., Clark, R.S.B., Jackson, E.K., and Dixon, C.E. (2015). Emerging therapies in traumatic brain injury. Semin. Neurol. 35, 83–100. 25714870 10.1055/s-0035-1544237 PMC4356170

[71] Voillet, V., Besse, P., Liaubet, L., San Cristobal, M., and González, I. (2016). Handling missing rows in multi-omics data integration: multiple imputation in multiple factor analysis framework. BMC Bioinformatics 17, 402. 27716030 10.1186/s12859-016-1273-5 PMC5048483

[72] Spearman, C. (1987). The proof and measurement of association between two things. By C. Spearman, 1904. Am. J. Psychol. 100, 441–471. 3322052

[73] Pituch, K.A., and Stevens, J. (2015). Applied Multivariate Statistics for the Social Sciences, 6th ed. Routledge/Taylor & Francis Group: New York.

