# Supplementary material for: Empowering Data Sharing and Analytics through the Open Data Commons for Traumatic Brain Injury Research
Source: Neurotrauma Rep. 2022 Apr 5;3(1):139–57. doi: 10.1089/neur.2021.0061 (PMC8985540; doi:10.1089/neur.2021.0061)

**SFigure 1.** Data and data dictionary formatting for ODC-TBI upload. (**A**) Experimental data is commonly recorded in spreadsheets with various structures designed to be human-readable including multiple tables and nested labels on a single spreadsheet. (**B**) ODC-TBI requires data to be formatted into the Tidy format. The first row contains the variable (i.e. column) names, and each column represents one of the dataset variables. Each corresponding row contains the values for an observation. (**C**) ODC-TBI allows the upload of a data dictionary with each dataset. The ODC-TBI data dictionary contains the following five columns: VariableName, Title, Unit_of_Measure, Description, and Comments.


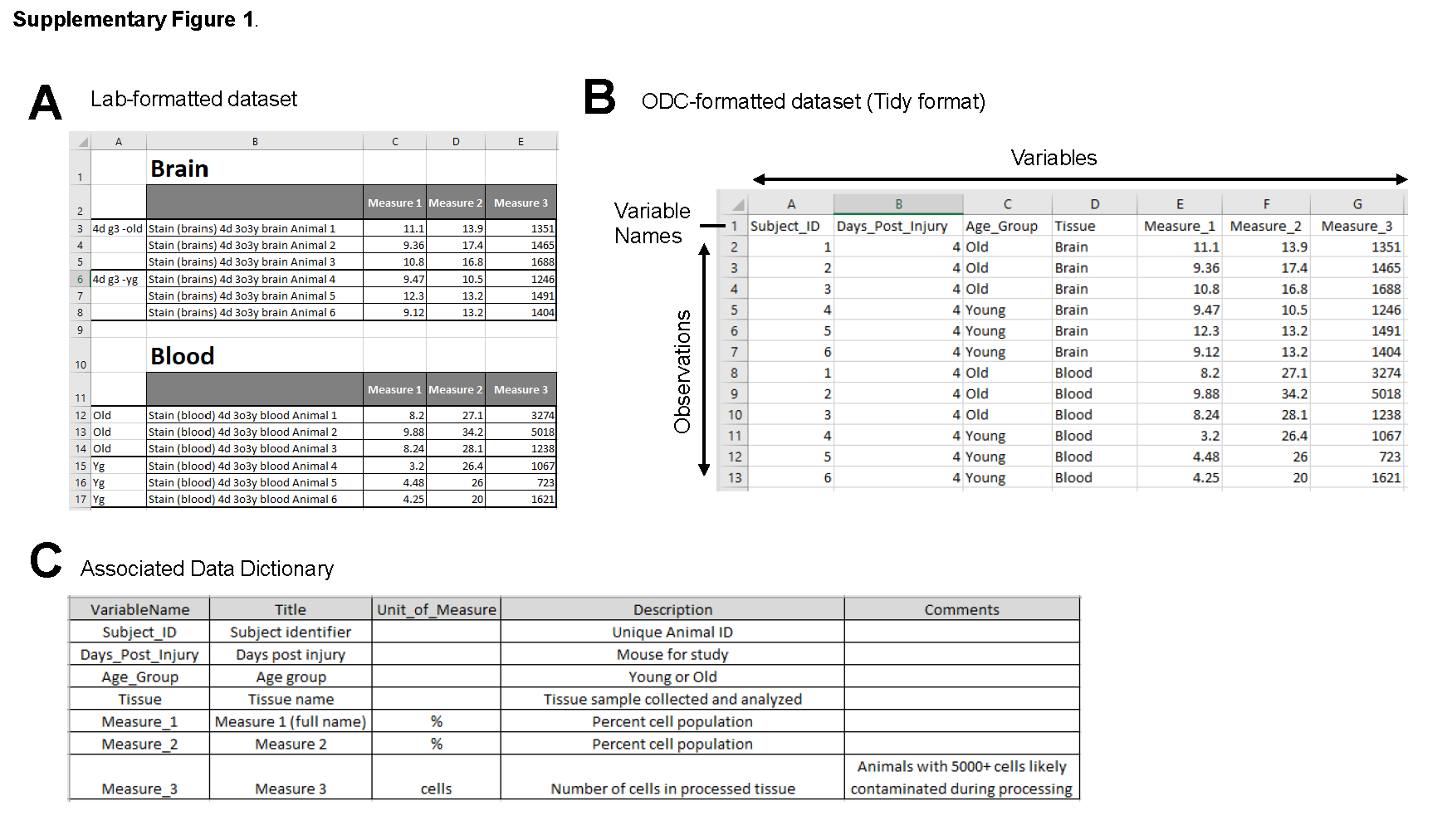

Supplement: Supplemental data [file Suppl_FigureS1.docx]
