# Supplementary material for: Empowering Data Sharing and Analytics through the Open Data Commons for Traumatic Brain Injury Research
Source: Neurotrauma Rep. 2022 Apr 5;3(1):139–57. doi: 10.1089/neur.2021.0061 (PMC8985540; doi:10.1089/neur.2021.0061)

**SFigure 2.** Heatmap representation of PCA. The heatmap visualization provides similar information as the barmap and shows all variable loadings including those below the threshold of significance (0.2) for each PC. The heatmap also shows the loadings for all PCs, including PC4, PC5, and PC6.


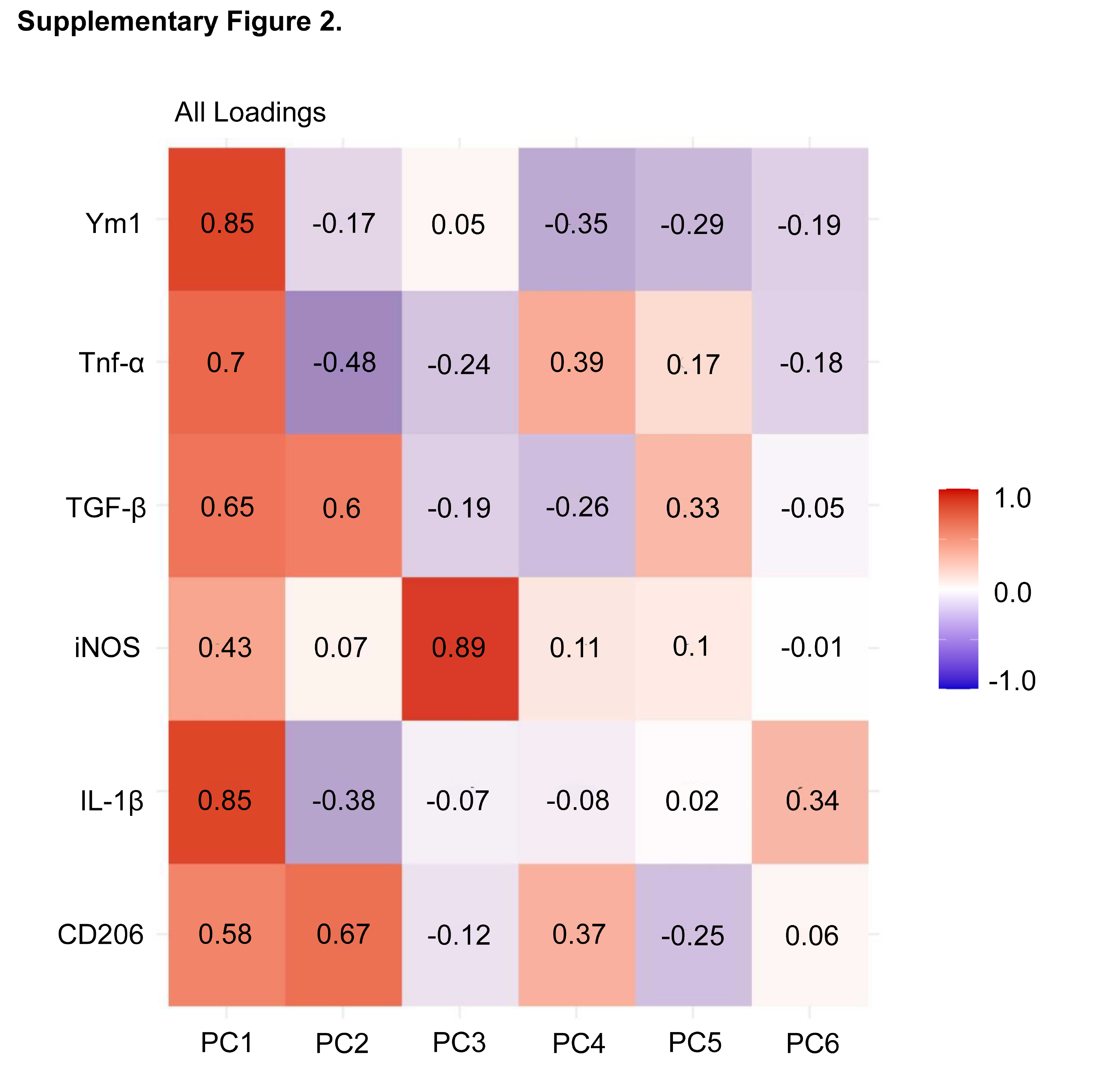

Supplement: Supplemental data [file Suppl_FigureS2.docx]
