# Supplementary material for: Empowering Data Sharing and Analytics through the Open Data Commons for Traumatic Brain Injury Research
Source: Neurotrauma Rep. 2022 Apr 5;3(1):139–57. doi: 10.1089/neur.2021.0061 (PMC8985540; doi:10.1089/neur.2021.0061)
Supplement: Supplemental data [file Suppl_TableS1.docx]

**Supplementary Table 1.** Effect size and observed power for Injury and Age effects for PCs and univariate inflammatory markers (sorted by descending effect size for each effect).

| Effect | Variable | Effect Size (η^2^) | Power |
| --- | --- | --- | --- |
| Injury | PC1 | 0.593513 | 1 |
|  | TGFB | 0.587202 | 1 |
|  | TNFa | 0.528806 | 0.999555 |
|  | IL1B | 0.38067 | 0.968824 |
|  | Ym1 | 0.37861 | 0.982232 |
|  | CD206 | 0.356279 | 0.999821 |
|  | PC2 | 0.088426 | 0.741933 |
|  | iNOS | 0.002289 | 0.05672 |
| Age | CD206 | 0.224005 | 0.992383 |
|  | PC2 | 0.222114 | 0.98516 |
|  | TGFB | 0.084818 | 0.789029 |
|  | Ym1 | 0.066147 | 0.397578 |
|  | iNOS | 0.051901 | 0.210149 |
|  | TNFa | 0.00562 | 0.084759 |
|  | PC1 | 0.002792 | 0.082704 |
|  | IL1B | 0.001879 | 0.058333 |
| Injury:Age | PC2 | 0.178517 | 0.959657 |
|  | CD206 | 0.146892 | 0.944496 |
|  | TGFB | 0.067209 | 0.691436 |
|  | Ym1 | 0.062637 | 0.380091 |
|  | iNOS | 0.024693 | 0.124739 |
|  | TNFa | 0.021873 | 0.189681 |
|  | PC1 | 0.011898 | 0.194191 |
|  | IL1B | 0.006931 | 0.081102 |
